# The impact of rare but severe vaccine adverse events on behaviour-disease dynamics: a network model

**DOI:** 10.1038/s41598-019-43596-7

**Published:** 2019-05-09

**Authors:** Samit Bhattacharyya, Amit Vutha, Chris T. Bauch

**Affiliations:** 1 Department of Mathematics, School of Natural Sciences, Shiv Nadar University, Greater Noida, India; 2 ICTS, Tata Institute for Fundamental Research, Bangalore, India; 3 Department of Applied Mathematics, University of Waterloo, Waterloo, Canada

**Keywords:** Ecological epidemiology, Computational models

## Abstract

The propagation of rumours about rare but severe adverse vaccination or infection events through social networks can strongly impact vaccination uptake. Here we model a coupled behaviour-disease system where individual risk perception regarding vaccines and infection are shaped by their personal experiences and the experiences of others. Information about vaccines and infection either propagates through the network or becomes available through globally available sources. Dynamics are studied on a range of network types. Individuals choose to vaccinate according to their personal perception of risk and information about infection prevalence. We study events ranging from common and mild, to severe and rare. We find that vaccine and infection adverse events have asymmetric impacts. Vaccine (but not infection) adverse events may significantly prolong the tail of an outbreak. Similarly, introducing a small risk of a vaccine adverse event may cause a steep decline in vaccine coverage, especially on scale-free networks. Global dissemination of information about infection prevalence boosts vaccine coverage more than local dissemination. Taken together, these findings highlight the dangers associated with vaccine rumour propagation through scale-free networks such as those exhibited by online social media, as well as the benefits of disseminating public health information through mass media.

## Introduction

Vaccination has been one of the most effective and cheapest measures to prevent infectious disease transmission since the discovery of smallpox vaccine in the year 1796 by English physician Edward Jenner^1^. Smallpox was one of the most devastating diseases in the history of humankind, but after implementing a worldwide mass vaccination program, the disease was certified by the World Health Organization (WHO) as being globally eradicated in 1980^2,3^. Today, most childhood infectious diseases are vaccine preventable, and can be controlled and eliminated by mass vaccination. Almost all countries have implemented some kind of routine childhood immunization program to control infectious diseases, with varying success^4–7^.

Individual vaccine decision-making is influenced by a range of factors, including historical, political, social, health and epidemiological^8–17^. A focus of mathematical models is often the epidemiological factors behind vaccine decision-making. For instance, disease dynamics–in particular, the generation of herd immunity and thus low disease incidence by high vaccine coverage–plays a role because individuals show less incentive to seek vaccination when they perceive a low risk of being infected^8^. The collective outcome of individual decisions not to get vaccinated reduces herd immunity, which may result in localized outbreaks^18^.

Perceived risk associated either with vaccines or the infections they prevent is also a driving factor in vaccine decision-making^8^. Despite the success of vaccines in controlling various infectious diseases and the strong safety profile of all modern vaccines, they are often perceived as risky by some members of the public^19–23^. When adverse health events are coincident with vaccination, individuals are prone to blame the vaccine^24–27^. For example, VAERS (Vaccine Adverse Events Reporting Systems, CDC and Food & Drug Administration, USA) received 128,717 case reports describing adverse events (such as fever, injection-site edema, rash, agitation, chest pain, vomiting, including paralysis and death) after immunization from January 1991 to December 2001^28,29^. During this period, 14.2% of all reports received were serious adverse events including deaths (1.4–2.3%), and life-threatening illness (1.4–2.8%). However, follow-up clinical and epidemiological investigation demonstrated strong evidence that vaccines were not the cause of these serious outcomes. The highest rates of reported adverse events were for rotavirus and pertussis vaccines (vaccines against childhood infectious diseases), and the lowest rates were for influenza and hepatitis B vaccines.

Online social media and mass media can serve to further disseminate perceptions of vaccine risks to the point that vaccine refusal can significantly impact vaccine coverage and herd immunity. For instance, significant declines in vaccine coverage caused outbreaks for the measles-mumps-rubella (MMR) vaccine “scare” in the 1990s as well as for polio in the 2000s^30–35^. The consequences of real or perceived adverse effects and changes in risk perception thus play a crucial role in the vaccine uptake in population^36^. Information about adverse events is often passed from one individual to another through their network of social contacts. Individual risk perception also depends on how individuals use information such as locally or globally available information to estimate their perceived risk. Therefore, when humans respond to the presence of a disease and make decisions that affect its control, we have a situation where both infectious pathogens and information about the pathogens spread simultaneously and interact with human decision-making^37^.

A significant body of literature explores models of the coupled dynamics of human and natural systems, for study systems ranging from land use to vaccinating behavior^38–51^. For instance, game theory has been integrated into epidemiological models to investigate vaccinating behaviour and analyze how different patterns in vaccine uptake and disease dynamics can emerge from simple assumptions^52–55^. Evolutionary game theory approaches that include more realistic aspects of decision-making such as social learning and social norms have also been developed^53^. Some research uses the framework of social contact networks to analyze the impact of heterogeneous contact patterns on individual vaccinating behavior^56–62^. However, most previous coupled behaviour-disease models simply assign a fixed cost to the decision to vaccinate, and do not distinguish between the probability of vaccine adverse events (whether common or rare) and their health impact (whether mild or severe).

Here, we explore these issues by developing a social network model of the coupled dynamics of infection spread, vaccinating behaviour, and individual risk perception due to adverse events, either from vaccination or infection. Adverse events are perceived to occur to a vaccinated or infected individual, and information about the event spreads to their neighbors through a contact network. To capture the ephemeral nature of information, the intensity of the information decays over time as it passes from neighbor to neighbor in a ripple effect. We model a self-limiting acute infection that can be prevented through vaccination and that spreads through the same contact network. Our objective is to understand how coupled behaviour-disease dynamics depend on the probability of adverse events either from vaccination or infection, and their respective severity. To observe the effect of contact patterns, we also consider different social networks such as regular lattice, random network, small world network, power law network, and an empirically-derived network. Our results suggest that a small probability of a severe adverse event may have an outsized impact on vaccine uptake and cumulative infections, depending on the contact pattern among individuals. Our analysis also indicates that global information has larger effect than local information, in terms of increasing vaccine coverage. In the next section, we describe the model.

## Methods

### Model and assumptions

We consider vaccination decision dynamics on a social network. We describe these aspects of the model structure in the following subsections: spreading of infection, spread of information on adverse events over the network that change the individual perceived risk, and individual vaccination decision-making.

#### Spreading of infection

We consider the standard SIR framework to model disease spread. The probability that a susceptible individual in the network acquires infection depends on the rate of disease transmission and the number of infected neighbors ${N}_{Inf}^{i}$. Let *β* be the transmission probability of infection. Therefore, at each time step, each susceptible *i* is infected with probability

1 $$Pro{b}_{Inf}=1-{(1-\beta )}^{{N}_{Inf}^{i}}.$$

An infected individual recovers and becomes immune with probability *γ* per time step.

#### Vaccination decision-making

We assume two strategies in the vaccination dynamics: vaccinator or non-vaccinator. Individuals can switch between these strategies. An individual’s vaccination decision is a function of payoffs for both strategies, i.e., perceived risk of infection and perceived benefits of vaccination. Suppose *X*_*i*_ indicates the strategy of individual *i* in the network, then

2 $${X}_{i}={\textstyle \{}\begin{array}{c}1,{\rm{i}}{\rm{f}}\,i\,{\rm{i}}{\rm{s}}\,{\rm{v}}{\rm{a}}{\rm{c}}{\rm{c}}{\rm{i}}{\rm{n}}{\rm{a}}{\rm{t}}{\rm{o}}{\rm{r}},\\ 0,{\rm{i}}{\rm{f}}\,i\,{\rm{i}}{\rm{s}}\,{\rm{n}}{\rm{o}}{\rm{n}}-{\rm{v}}{\rm{a}}{\rm{c}}{\rm{c}}{\rm{i}}{\rm{n}}{\rm{a}}{\rm{t}}{\rm{o}}{\rm{r}}\end{array}$$

We assume that every individual *i* has their own level of perceived vaccine risk *C*_*V*,*i*_ and perceived infection risk *C*_*I*,*i*_. The values of these may change over time according to rules discussed in the following subsection. If *P*_*V*,*i*_ is the perceived payoff of an individual *i* who is a vaccinator and *P*_*NV*,*i*_ is the perceived payoff for non-vaccinator, then

3 $$\begin{array}{rcl}P({X}_{i}=1) & = & {P}_{V,i}=-\,{C}_{V,i},\\ P({X}_{i}=0) & = & {P}_{NV,i}=-\,{C}_{I,i}{\theta }_{i}\end{array}$$

where *θ*_*i*_ is the perceived probability of infection (also discussed below). The individual decision to become a vaccinator is motivated by maximizing the payoff. We also assume that individuals switch strategies according to the Fermi-Dirac function, such that the probability of individual *i* switching to vaccinator is:

4 $${{\rm{\Phi }}}_{i}({\rm{\Delta }}{P}_{i})=\frac{1}{1+\exp (\,-\,\xi {\rm{\Delta }}{P}_{i})},$$

where Δ*P*_*i*_ = *P*(*X*_*i*_ = 1) − *P*(*X*_*i*_ = 0) is the payoff gain of node *i* given by Δ*P*_*i*_ = −*C*_*V*,*i*_ + *C*_*I*,*i*_*θ*_*i*_. For example, if Δ*P*_*i*_ > 0, the node *i* will vaccinate with probability Φ_*i*_(Δ*P*_*i*_). When Δ*P*_*i*_ = 0, the individual *i* will vaccinate or not vaccinate with equal probability. The parameter *ξ* determines the individual responsiveness to payoff differences. The equation (4) known as the Fermi function has been widely used to decision-making models^63^. For low values of *ξ*, equation (4) changes more gradually as *P*_*i*_ goes from negative to positive, meaning that individuals are less responsive to the payoff differences. However, for high values of of *ξ* in equation (4) indicates that individuals are highly responsive to the payoff differences.

#### Adverse events and spread of the information

Adverse events may occur either from vaccination or infection. We define an infection adverse event as a significant health outcome perceived to arise from an infection (such as hospitalization for pneumonia). Similarly, an vaccine adverse event is a significant health outcome perceived to arise from becoming vaccinated (such as experiencing a severe flu-like illness). Individuals use their perception of risk in their decision-making rather than the actual risk, meaning that they may misattribute certain experiences to the infection or the vaccine. Individuals experience an adverse event upon infection or vaccination with some probability. The information about an individual’s adverse events due to infection or vaccine propagates throughout the network and influences the perceived risks of neighboring nodes. We assume the intensity of the information decays as it propagates through the network from one node to its neighboring nodes, much like ripples in a pond. Hence, individuals take in information from all over the network, although it is attenuated depending on how far they are from the individual who experienced the adverse event. The perceived risks *C*_*V*,._ and *C*_*I*,._ change as per the following rules:

(i) Whenever individual *j* gets vaccinated, there is a probability *α*_*V*_ of an event. In this case, their value of *C*_*V*,*j*_ is increased by an amount *κ*_*V*_. Their neighbour’s value of *C*_*V*,*j*_ is increased by an amount *κ*_*V*_ ∗ *ω* where 0 < *ω* < 1. Their neighbour’s neighbour’s value of *C*_*V*,*j*_ is increased by an amount *κ*_*V*_ ∗ *ω*^2^, *etc*.
(ii) Whenever individual *j* gets infected, there is a probability *α*_*I*_ of an infection adverse event. In this case, their value of *C*_*I*,*j*_ is increased by an amount *κ*_*I*_. Their neighbour’s value of *C*_*I*,*j*_ is increased by an amount *κ*_*I*_ ∗ *ν* where 0 < *ν* < 1. their neighbour’s neighbours value of *C*_*I*,*j*_ is increased by an amount *κ*_*I*_ ∗ *ν*^2^, etc.

The perceived probability of infection *θ*_*j*_ in equation (3) for individual *j* depends on information about global prevalence of infection versus local prevalence in their network neighborhood. We define *θ*_*j*_ as follows:

5 $$\begin{array}{rcl}{\theta }_{j} & = & \rho \frac{\,\#\,{\rm{local}}\,{\rm{infections}}\,}{\,\#\,{\rm{all}}\,{\rm{infected}}\,{\rm{in}}\,{\rm{the}}\,{\rm{population}}\,}\\  &  & +(1-\rho )\frac{\,\#\,{\rm{global}}\,{\rm{infections}}}{\,\#\,{\rm{all}}\,{\rm{infected}}\,{\rm{in}}\,{\rm{the}}\,{\rm{population}}},\end{array}$$

where *ρ* denotes the relative importance of local vs. global information. The demarkation of local and global infection depends on the neighborhood size *n*, where *n* = 1 denotes immediate neighbors of individual *j*, *n* = 2 implies neighbors’ neighbors, and so on.

### Simulations

#### Contact network

We analyze the effect of adverse events on vaccination dynamics across different network types. Our baseline analysis uses a power-law (scale-free) network but we also explore model predictions using a lattice, a small-world network, an Erdos-Renyi random network, and an empirically-derived network. All these networks have similar node numbers ~5000. The degree distribution of the power-law network is given by *Ck*^−2.7^ with average degree 2. The average degree of the Erdos-Renyi random network is 31 and the average degree of the small world network is 12 (Figure S1). Contact networks were generated using the *igraph* software package for complex network research^64^. Scale-free networks were generated using the Barabasi-Albert preferential attachment algorithm^65^. Random networks of varying average degree were generated using the Erdos Renyi *G*(*n*, *p*) model, with values of *p* varied to produce varying average degrees^66^. Small world networks were generated using the Watts-Strogatz algorithm^67^. We used five contact networks of 10,000 nodes each, obtained by sampling subnetworks from a large contact network derived from empirical contact patterns in Portland, Oregon^68–70^. The average degree for all five network lies in the range 75–80 (Figure S1). The properties of these networks are described in more detail in ref.^71^.

#### Simulation Algorithm

First, we describe the simulation algorithm in the absence of any vaccination, which allows us to calibrate the transmission parameters. Here, we assume that individual nodes can be in one of three mutually exclusive states: Susceptible, Infected, or Recovered. The steps in the algorithm are as follows:

1. Generate the contact network and assign all individuals as susceptible.
2. Randomly select a finite number of individuals and assign them to the infected state.
3. Infect susceptible individuals by infected neighbors with probability given by equation (1) and, recover infected individuals with probability *γ* per time step.
4. Introduce demographic events (i.e., birth) by making a random node susceptible with probability 0.004 per capita per year. To protect from stochastic extinction of infection, a very minimal level of case importation is considered by making a random susceptible node as infected with probability 0.002 per capita per year.
5. Repeat steps 5–8 until the system shows no new infections in three consecutive steps.

We thus simulate the transmission of disease and calibrate epidemic parameters to ensure that infection risk in an unvaccinated population is equal across the network types. Once this baseline, vaccine-free scenario has been established, we introduce vaccination decisions for individuals (nodes) in the network. Here, we assume that individuals can have Vaccinated status along with the other three status. The steps of the simulation algorithm for epidemic scenarios in presence of vaccination are as follows:

1. Generate the contact network and assign all individuals as susceptible.
2. Randomly select a finite number of individuals and assign them to the infected state.
3. Compute *θ*_*j*_, the perceived probability of infection for each individual in the network in the initial time-step.
4. Randomly assign each individual a perceived vaccination risk *C*_*V*,*j*_ and perceived infected risk *C*_*V*,*j*_, by uniformly sampling the unit interval (0, 1). Each individual acquires the status of vaccination according to its vaccination strategy, except vaccinated individuals cannot become unvaccinated. Vaccinated individuals are immune to infection.
5. Compute the payoffs for each individual, and use the Fermi-Dirac form of the decision function to decide whether individuals choose the vaccinator strategy (Equation 4).
6. Trigger adverse infection and vaccination events by randomly sampling the unit interval with probabilities *α*_*V*_ and *α*_*I*_ respectively. Update perceived risks for all individuals in the network based on the distance of a given individual from the source of the adverse event.
7. Infect susceptible individuals by infected neighbors with probability given by equation (1) and, recover infected individuals with probability *γ* per time step.
8. Introduce demographic events (i.e., birth) by making a random node susceptible with probability 0.004 per capita per year. To protect from stochastic extinction of infection, a very minimal level of case importation is considered by making a random susceptible node as infected with probability 0.002 per capita per year.
9. Repeat steps 5–8 until the system shows no new infections in three consecutive steps.

We calibrate the choice and cost parameters and again scale baseline values such that major disease outbreak expands for around 3–4 months in absence of vaccination (see result). When vaccination is present in the system, we calibrate base values of different parameters so that it attains high vaccination coverage in the absence of adverse events. Next, we introduce adverse events and again check their transient effects on vaccination decision dynamics part of the model calibration. The baseline parameter values resulting from this calibration exercise (Table 1) are used unless stated otherwise. These baseline parameter values could correspond to an outbreak of influenza in a population with access to a well-matched vaccine and who may choose to vaccinate during the outbreak, as occurred during the 2009 H1N1 pandemic for example. The equilibrium results represent the averages over 100 independent iterations of steps 5–9 in the algorithm. The simulation code was written in the C++ programming language and appears in supplementary information as Methods S1.

**Table 1:** Baseline parameter values used in the simulation of model.

Parameter	Description	Value, Range
*β*	disease transmission probability	0.6, calibrated
1/*γ*	infectious period	3 days
*α*_*V*_	probability of adverse event from vaccination	parameter of interest, (0, 1)
*α*_*I*_	probability of adverse event from infection	parameter of interest, (0, 1)
*κ*_*V*_	Increment in perceived vaccine risk	0.6, (0, 1)
*κ*_*I*_	Increment in perceived infection risk	0.7, (0, 1)
*ρ*	Weight of Local-Global information spread	parameter of interest, (0, 1)
*ω*	Information spread coefficient, vaccine adverse events	0.8, (0, 1)
*ν*	Information spread coefficient, infection adverse events	0.8, (0, 1)
*L*	Baseline payoff	1
*ξ*	Degree of responsiveness to differences of payoff	6, (2–20)
1/*μ*	Life expectancy	75 years
*n*	Local neighborhood size	parameter of interest, (1, 10)
*A*_*m*_	Time window during which adverse event may occur	2 days

## Results

We explore the effect of adverse events either from vaccines or infections on the individual vaccination decisions. We also explore the intensity of the effect of adverse events and decisions depend on whether individuals use local or the global information to estimate the perceived risk from infection.

### Effect of adverse events

**Figure 1:**
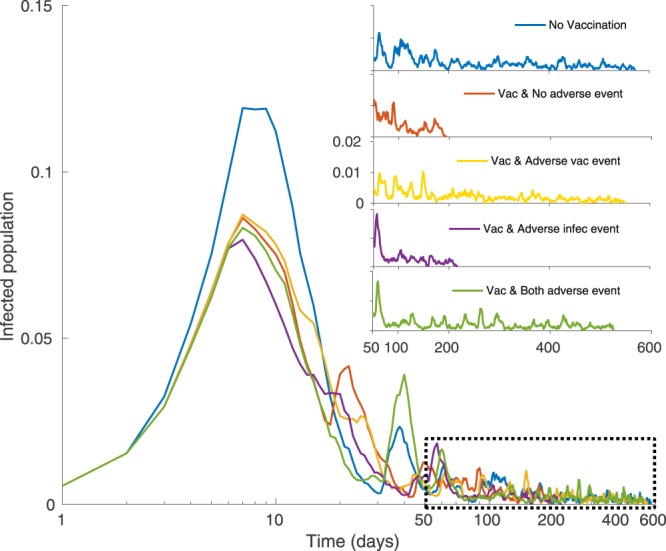
A sample time series of the model simulation showing the proportion of infected persons over time (days). Each colour represents a different setting of the model: no vaccination allowed (blue); vaccination allowed, but no adverse events (red); vaccination and adverse vaccination events allowed (yellow); vaccination and infection adverse events allowed (purple); vaccination and both vaccine and infection adverse events allowed (green). Parameter values were *α*_*I*_ = 0.01 = *α*_*V*_, *ρ* = 0.6, and *n* = 5, with other parameter values as in Table 1. The same random number seed was used for all scenarios.

The impact of vaccine adverse events on model dynamics is highly apparent in the tail of simulated outbreaks, but less apparent in the bulk of the epidemic curve (Fig. 1). When vaccination is introduced to the population, simulations–with or without the possibility of adverse events from either vaccines or infection–behave in similar ways for the bulk of the epidemic curve: vaccination reduces the epidemic peak to a similar degree whether or not adverse vaccine or infection events are included (although the peak is somewhat lower when infection adverse events are included, as expected). However, epidemics in the presence of vaccine adverse events–with or without the presence of infection adverse events–last 150% longer (300 more days) than epidemics in the absence of vaccine adverse events. This finding is robust across multiple stochastic realizations at the same parameter values: the average duration of the epidemic when vaccination is allowed is 168 ± 117 days in the absence of both vaccine and infection adverse events, compared to 272 ± 166 days in the presence of vaccine adverse events, or 303 ± 147 days in the presence of both infection and vaccine adverse events (Figure S2). Interestingly, the duration of the epidemic is lengthened–not shortened–when infection adverse events are introduced. For instance, the epidemic duration is 168 ± 117 days in the absence of both vaccine and infection adverse events, compared to 213 ± 143 days in the presence of infection adverse events (but not vaccine adverse events). This long epidemic tail is due to pockets of unvaccinated individuals who continue to fuel the outbreak in its later stages. The size of epidemic peak and length of epidemic tail vary depending on whether individuals are using global or local information to determine their perceived infection probability (Figure S3). Also, the proportion of cumulative vaccination coverage depends on the dynamics of adverse events (Figure S4). For example, cumulative vaccination vaccination coverage is highest when there are only adverse infection events, and but it is lowest when there are adverse events from vaccination. This phenomenon suggests that the circulation of stories about vaccine adverse events in the population not only makes it difficult to achieve elimination due to herd immunity effects, as suggested by analyses of endemic disease states^52,53^, but it may also significantly prolong any given outbreak of a vaccine-preventable infectious disease.

Our baseline model assumes local spread of information about adverse events, since our objective was to study how information (whether true or false) that spreads through social networks can influence vaccinating behaviour. However, the case of global spread can be obtained in our model as a special case *ω* = *ν* = 1 (in this case, the information from a single event spreads throughout the network instantaneously, without a decay in its impact). Simulations of this special case emphasize the starkly differing patterns that occur for local versus global spread of information about adverse events (Figure S5). For instance, compared to the baseline scenario of Fig. 1 where dissemination of adverse events is local, we find that the epidemic peak is significantly lower in the presence of global dissemination of adverse infection events, with or without the presence of adverse vaccine events. Hence, global dissemination of adverse infection events (such as through mass media) may be a useful public health strategy.

In some cases, it has been observed that individuals show a much stronger reaction to spectacular rumours about vaccine adverse events than infection adverse events, and so, the news about the vaccine adverse event may spread more widely through social networks. To quantify the effect of this on model dynamics, we simulate the model under different values of *ω* (the spreading coefficient of vaccine adverse event information) when *ν* = 0.25 and *ν* = 0.5 (Figure S6). At both values of *ν*, it is observed that the cumulative vaccine coverage and infection incidence are relatively unresponsive to changes in *ω* when *ω* < 0.5, but beyond that point the vaccine coverage starts decreasing and infection incidence starts increasing steadily, as *ω* increases. These results show that the difference in how individuals pass on information about vaccines or infection can have nontrivial effects on population-level outcomes.

**Figure 2:**
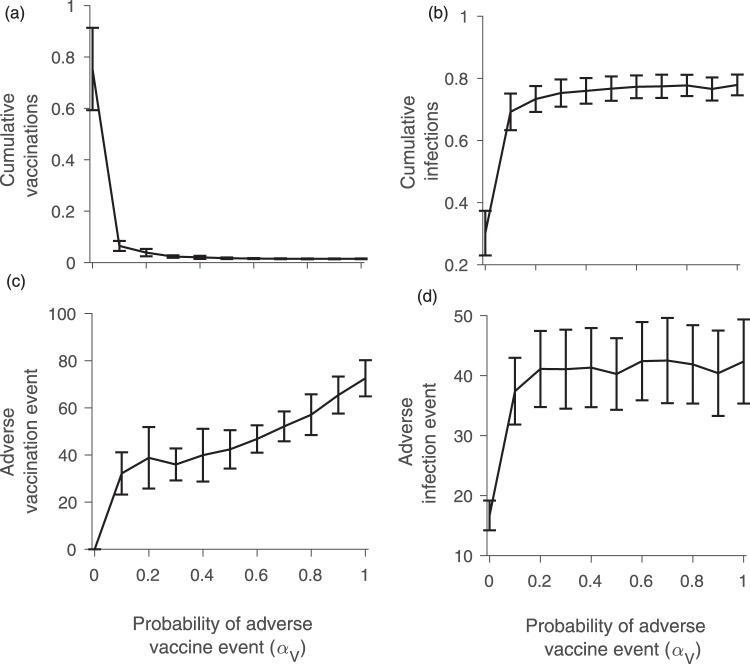
Figure shows (**a**) cumulative proportion vaccinated; (**b**) cumulative proportion infected; (**c**) number of adverse vaccination events; and (**d**) number of infection adverse events under different values of the probability *α*_*V*_ of a vaccine adverse event. The error bars show two standard deviations for 100 simulations conducted for each point on the plot. Parameter values were *α*_*I*_ = 0.01, *ρ* = 0.6, and *n* = 5, with other parameter values as in Table 1.

**Figure 3:**
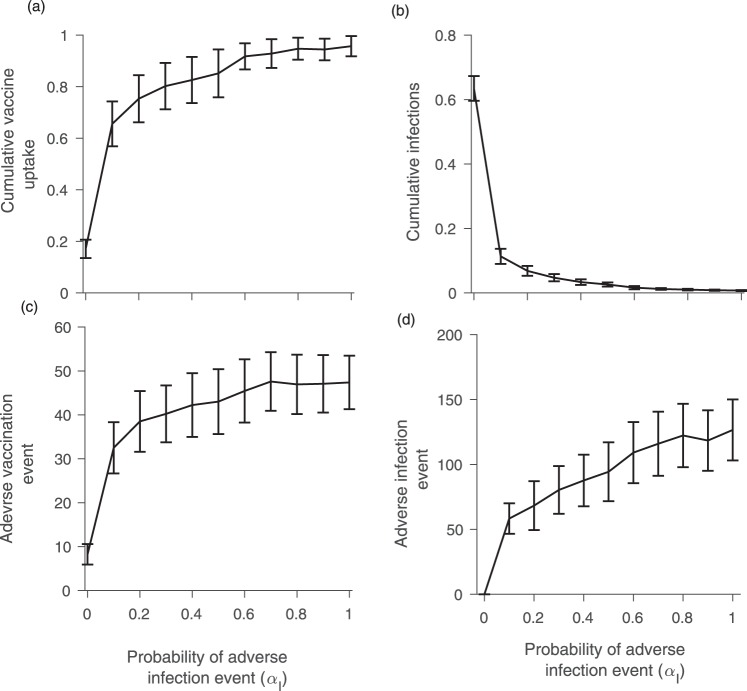
Figure shows (**a**) cumulative proportion vaccinated; (**b**) cumulative proportion infected; (**c**) number of adverse vaccination events; and (**d**) number of infection adverse events under different values of the probability *α*_*I*_ of an infection adverse event. The error bars show two standard deviations for 100 simulations conducted for each point on the plot. Parameter values were *α*_*V*_ = 0.01, *ρ* = 0.6, and *n* = 5, with other parameter values as in Table 1.

Model simulations show an asymmetry in how behaviour responds to changes in the probabilities of vaccine adverse events versus disease adverse events. Only small increases in the probabilities of vaccine adverse events *α*_*V*_ and infection adverse events *α*_*I*_ from zero are required to have a large impact on the cumulative vaccination coverage, cumulative infections, and number of vaccine and infection adverse events (Figs 2 and 3). However, these four outcomes react very differently to changes in *α*_*V*_ compared to changes in *α*_*I*_. As *α*_*V*_ is increased just slightly above zero, vaccine coverage drops very steeply, falling to approximately 6% at *α*_*V*_ ≈ 0.1 (Fig. 2a). Further increases in *α*_*V*_ cause only slight continuing declines in the vaccine coverage. This nonlinear response is also reflected in the cumulative number of infections, which increases suddenly as *α*_*V*_ becomes nonzero and thereafter levels off with growing *α*_*V*_ (Fig. 2b). The dependence of the number of adverse vaccine and infection events on *α*_*V*_ reflects trends observed for vaccine coverage and number of infections (Fig. 2c,d). These four outcomes react to changes in the infection adverse event probability *α*_*I*_ in a similar way, but the rise in vaccine coverage as *α*_*I*_ increases above zero is somewhat less steep (Fig. 3a). However, it is worth noting that the dependence of these outcomes on *α*_*V*_ is more gradual when *α*_*I*_ is (unrealistically) large, and *vice versa* (see parameter planes in Figure S7). The asymmetric response of the model to *α*_*V*_ versus *α*_*I*_ reflects differing conditions for vaccine adverse events versus infection adverse events. In a context of low infection incidence (and therefore very few infection adverse events) even a small probability of vaccine adverse events can cause a significant decline in vaccine uptake. However, increasing the probability of infection adverse events in the context of an already endemic disease causes a more proportionate and gradual increase in vaccine coverage since the system is already far from the elimination threshold. Taken together, these results show that a small probability of adverse events can dramatically change individual vaccine decision-making and epidemic outcomes.

**Figure 4:**
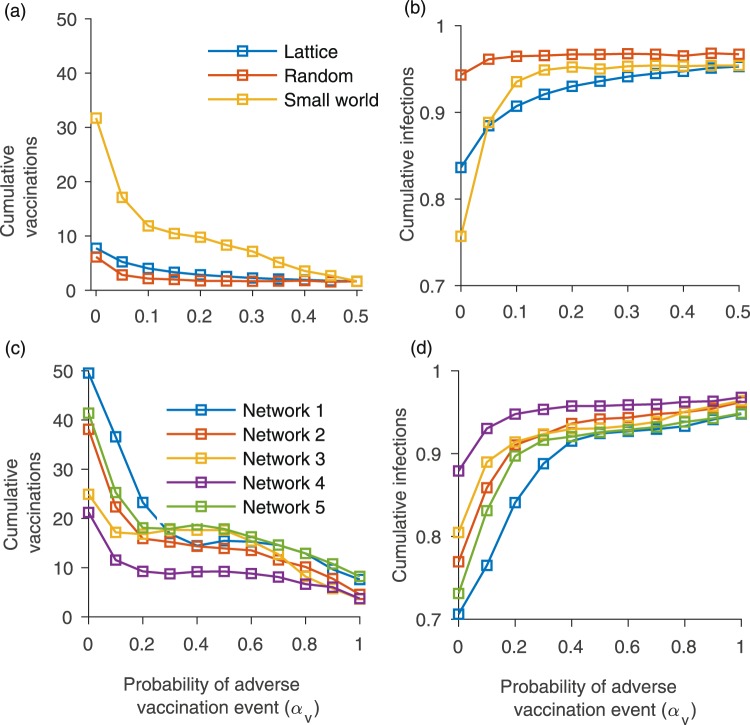
Figure shows (**a**) cumulative proportion vaccinated and (**b**) cumulative proportion infected for different values of the probability *α*_*V*_ of an adverse vaccination event, for a regular lattice, random network, and small world network with similar numbers of nodes. Subpanels (**c**) and (**d**) are same, but use five different empirically-derived networks. Details of the networks are given in the Methods. The error bars show two standard deviations for 100 simulations conducted for each point on the plot. We consider *α*_*I*_ = 0.01, *ρ* = 0.6, and *n* = 5, with other parameter values are as in Table 1.

These simulations (Figs 2 and 3) were conducted on a scale-free contact network. Hence, we also explore how cumulative vaccine coverage and infections depend upon *α*_*V*_ for a regular lattice, small-world network, random network, and empirically-derived networks (Fig. 4). These other network types show a more gradual response of model dynamics as *α*_*V*_ increases from zero. This suggests that strong changes in model dynamics for small probabilities of adverse events observed in Figs 2 and 3 are a function of network structure and not only the mechanism of decision-making *per se*. Of the alternative network types, the dependence of vaccine coverage and infections on *α*_*V*_ is closest to linear for the regular lattice, perhaps on account of its homogeneous structure: all individuals have the same number of contacts and hence everyone experiences adverse events in their neighbourhood in a similar way. The dependence of vaccine coverage on *α*_*V*_ is somewhat less gradual for the small-world, random and empirical networks, on account of their more variable neighbourhood size. And, as already observed, the dependence is strong for the scale-free network on account of its highly skewed node degree distribution (Figs 2 and 3). The comparison of model dynamics for different network types shows that effect of the adverse events on vaccination decisions and hence on disease dynamics depends on the contact topology of individuals in the population.

### Effect of local versus global information about infection cases

Models provide a useful way of comparing the effects of local versus global transmission of information about cases of infection, since it is difficult to experimentally manipulate where individuals get their information from in real study populations. In our model, *ρ* governs whether individuals receive information about the number of infections in the population globally, from the number of cases in the entire population, or locally, from the number of infections in their network neighbourhood. Also, individuals look at *n*^*th*^ order neighbours to find information about infected cases. For instance, when *n* = 2, individuals count the number of infected cases in their neighbours and their neighbour’s neighbours.

**Figure 5:**
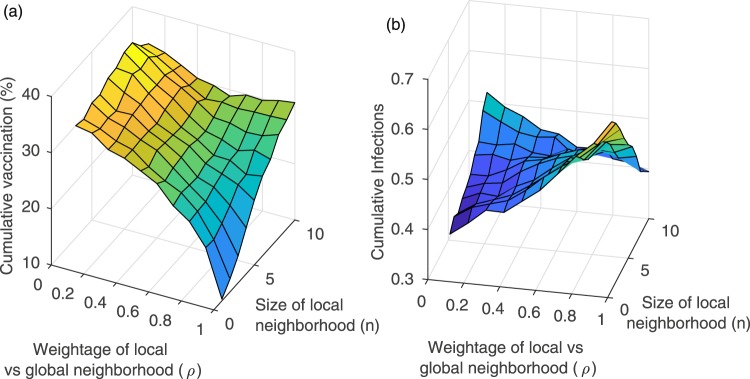
(**a**) Cumulative proportion vaccinated and (**b**) cumulative proportion infected under different values of size (*n*) of local neighborhood and weightage (*ρ*) of local vs. global neighborhood. *α*_*V*_ = 0.01 = *α*_*I*_ with other parameter values are as in Table 1.

Analysis of how the interplay between *ρ* and *n* determines cumulative vaccine coverage suggests that individuals obtaining information from global sources may be optimal from a public health perspective. We investigated how cumulative vaccine coverage depends on *ρ* and *n* (Fig. 5). When *ρ* is large and thus individuals get much of their information about infections locally, an increase in *n* can increase vaccine coverage. This occurs because when individuals pay attention to a larger local neighbourhood of the network, they will see more infections, which in turn stimulates vaccine uptake by making the vaccinator payoff more attractive. Using larger neighbourhoods also allows individuals lead time to prophylactically vaccinate before the infection reaches their first-order neighbours. However, the increase in vaccine uptake as a function of *n* is relatively inefficient. For instance, when *ρ* = 1, an increase from *n* = 1 to *n* = 5 causes vaccine uptake to increase from 10% to ≈25%, but *n* = 5 is a very generous fifth-order neighbourhood size. It is possible to increase vaccine coverage still further by moving from *n* = 5 to an impractical *n* = 10, but this only increases vaccine coverage another 10%. In contrast, when individuals simply get all of their information globally (*ρ* = 0), vaccine coverage is higher (and does not depend significantly on *n*, as expected). This suggests that obtaining information from global sources may be optimal, both in terms of maximizing vaccine coverage as well as in terms of what is more practical compared to obtaining information from chains of neighbours.

We also explored the dynamics in the *ρ* and *n* parameter space for the other network types. The impact of local-global information depends to some extent on network type although our overall findings are the same. We found qualitatively similar dynamics, except that the transition to higher vaccine coverage is less continuous and happens at a lower neighbourhood size *n* for the small-world and random networks, although a third- or fourth-order neighbourhood size is still required for high vaccine coverage (Figure S8). For the regular lattice in the case of large *ρ*, the dependence of vaccine coverage on *n* is almost completely flat.

Figures 2 and 3 showed a strong dependence of vaccine coverage on small adverse event probabilities. We explored the impact of changing *ρ* on this outcome as well, finding that small increases in *α*_*V*_ from zero continue to cause a steep decline in vaccine coverage regardless of whether *ρ* is low or high (Figure S9a,b), although the vaccine coverage for higher *α*_*V*_ is relatively higher when individuals use global information. Finally, we explored an alternative functional form for how individuals use information about infections in their local neighbourhood to assess their infection risk. Instead of Equation 5 we used the formulation

6 $$\begin{array}{rcl}{\theta }_{j} & = & \rho \frac{\#\,{\rm{local}}\,{\rm{infected}}\,{\rm{neighbors}}}{\,\#\,{\rm{all}}\,{\rm{local}}\,{\rm{neighbors}}}\\  &  & +\mathrm{(1}-\rho )\frac{\,\#\,{\rm{all}}\,{\rm{infected}}\,{\rm{in}}\,{\rm{the}}\,{\rm{population}}}{\,{\rm{Total}}\,{\rm{population}}\,}\end{array}$$

As in Figs 2 and 3, we continue to observe a rapid decline in vaccine uptake upon introducing a small nonzero probability *α*_*V*_, and regardless of whether *ρ* = 0 or *ρ* = 1 (Figure S9c,d). Figure S9 shows substantial variability in the difference of vaccination coverage for *ρ* = 1 and *ρ* = 0 and corresponding infections as a result of local vs global information spread.

### Tradeoffs between severity and probability of adverse events, and range of information spread

**Figure 6:**
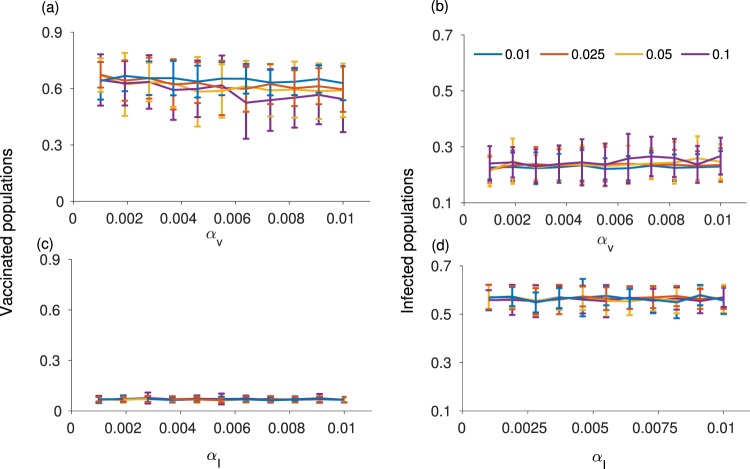
Figure shows the (**a**) cumulative proportion vaccinated and (**b**) cumulative proportion infected, when the product *α*_*V*_*κ*_*V*_) of the probability of a vaccine adverse event (*α*_*V*_) and its severity (*κ*_*V*_) remain constant (values of the product are indicated by the legend). Although there is little change in the average vaccination level as the value of the product increases, there is large variability in vaccine coverage. (**c**) and (**d**) show the same when the product (*α*_*I*_*κ*_*I*_) of the probability (*α*_*I*_) of an infection adverse event and its severity (*κ*_*I*_) remains constant. There is not much change in the average level, but large variability exists in both vaccinated and infected populations.

We also investigated the relationship between the probability and severity of adverse events, and how far information about those events spreads. First we explored whether model dynamics under a high probability of mild adverse events are similar to model dynamics under a low probability of severe adverse events. We explored simulation results across a range of values for *α*_*V*_ such that *α*_*V*_ × *κ*_*V*_ is a constant (where *κ*_*V*_ is the severity of vaccine adverse events). We did a similar exercise for *α*_*I*_ such that *α*_*I*_ × *κ*_*I*_) is constant. We found that vaccine coverage declines only slightly with increasing *α*_*V*_ (Fig. 6(a)) and is essentially unchanged with increasing *α*_*I*_ (Fig. 6(c)). This result reflects our model assumption that the individual payoff depends (implicitly) on a multiplicative product of the adverse event probability and the severity of the event, such that doubling the number of adverse events has the same average impact as doubling the severity of adverse events.

The variability between outcomes of different stochastic realizations is also unchanged as *α*_*V*_ increases (Fig. 6). This might reflect that rare but severe adverse events–while relatively uncommon in the population–propagate further in the network on account of their great severity (higher *κ*_*V*_ and *κ*_*I*_ values). In contrast, information about commonplace events that are mild propagate less far in the network, but the event occurrences are also more widely distributed. Hence, the average effects of rare but severe events are similar to those of common but mild events in our highly controlled simulation experiment (see our Discussion section on the potential impacts of prospect theory and other probability distributions, however).

**Figure 7:**
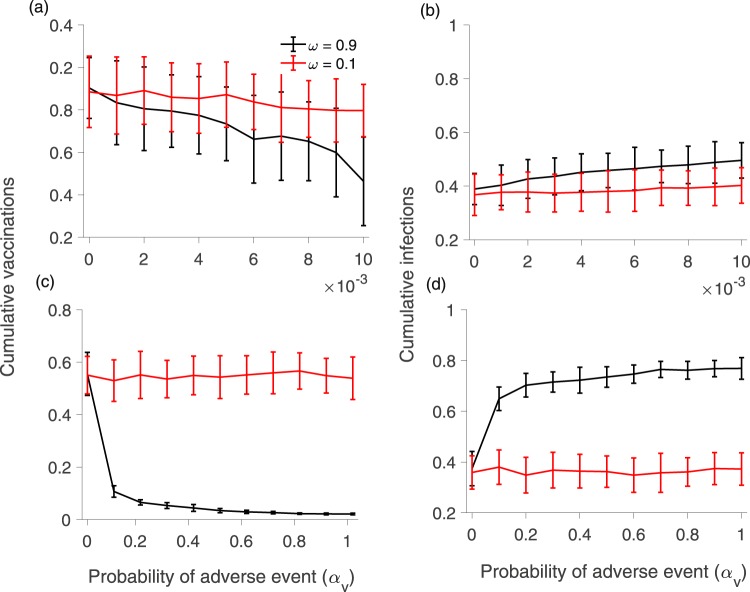
Figure shows cumulative proportion vaccinated (**a**,**c**) and cumulative proportion infected (**b**,**d**) under different values of probability of vaccination event *α*_*v*_ when there are severe but rare events that spreads globally (**a**,**b**: black lines), compared to when there are frequent and mild adverse events that spreads locally (**c**,**d**: red lines). The other parameter values are as in Table 1, except *κ*_*v*_ = 1.5, and *ω*_*v*_ = 0.9 for the upper panels and *κ*_*v*_ = 0.1, and *ω*_*v*_ = 0.1 for the lower panels. We also plot counterfactuals for each case for comparison (*ω*_*v*_ = 0.1 for the top panel and *ω*_*v*_ = 0.9 for the bottom panel).

To further explore the interaction between range and severity, we contrasted model dynamics for a rare and severe vaccine adverse event that spreads far across the network (low *α*_*v*_, high *κ*_*V*_, high *ω*) and a common and mild vaccine adverse event that spreads only locally (high *α*_*v*_, low *κ*_*V*_, low *ω*) (Fig. 7). This scenario captures the observation from real populations that rare but frightening adverse events can be disseminated more widely through social networks than mild and commonplace events. The results show that rare but severe events that spread far across network cause a significant reduction in vaccination coverage (Fig. 7a,b, black lines), compared common adverse events that occur very frequently but do not spread very far (Fig. 7c,d, red lines). The former cause a decline in vaccine coverage as *α*_*V*_ increases, while vaccine coverage does not decline at all as *α*_*V*_ increases, in the latter. The outsized importance of rare events is shown by comparing utility function impacts of the two types of adverse events, as follows. When *α*_*V*_ = 0.01 in the case of a rare but severe adverse event (Fig. 7a,b), we have that *α*_*V*_ × *κ*_*V*_ = 0.015 as the impact on the utility function. In contrast, when *α*_*V*_ = 1 in the case of a mild but common adverse event (Fig. 7c,d), we have that *α*_*V*_ × *κ*_*V*_ = 0.1. The penalty imposed on the payoff function of an individual who experiences the mild event is much larger than what is imposed by a severe event (0.1 versus 0.015), but because the information about the severe event spreads further, it has a greater impact on individual behaviour and cumulative vaccine coverage.

For comparison, we also display the outcomes for the opposite scenarios for events that are rare, severe, but do not spread far (Fig. 7a,b, red lines) and events that are common, mild and do spread far (Fig. 7a,b, black lines). In this (counterfactual) case, vaccine coverage declines dramatically with an increasing adverse event probability for the mild events that spread far, but not the severe events that spread locally. This emphasizes the importance of the parameter governing how far the information about the adverse events spreads through the social network. To further explore this counterfactual case, we compare a case of high *κ*_*V*_ and low *ω* to a case of low *κ*_*V*_ and high *ω*. As expected, we find that there is no significant change in the vaccine coverage across different probability of adverse event in case of higher *κ*_*V*_ and lower *ω*, but vaccinated population steeply declines when *κ*_*V*_ is lower and *ω* is high (Figure S8).

## Discussion

Many theoretical models of coupled behaviour-disease interactions represent a world where individuals mix homogeneously, where individuals all have uniform knowledge of vaccine and infection risks and impacts, and where those risks and impacts are represented by a single cost parameter in a utility function. For many applications this can be a useful approximation. However, rare but severe (real or perceived) adverse events associated with vaccines or infections can alter population vaccinating behaviour in different ways from common but mild adverse events. Moreover, news of these events may travel through online social media networks instead of through mass communication. In order to address these issues in a theoretical modelling framework we developed a social network simulation model of coupled behaviour-disease dynamics. We studied the influence of severity and rarity of adverse events on vaccinating decisions and disease dynamics during an outbreak, and how the outcomes depend on other features such as network structure and local versus global dissemination of information about adverse events. Each individual can have a different perception of vaccine and infections risks that is shaped by their experience and the experiences shared by neighbours, or globally disseminated information.

Some of our findings are relevant to population vaccinating behaviour and public health interventions, especially in the age of online social media. For instance, we observed that vaccine adverse events, unlike infection adverse events, can cause epidemic outbreaks of vaccine-preventable infections to have a very long tail (Fig. 1). This necessitates a longer and more drawn out public health intervention. Moreover, a population undergoing such a dynamic represents a continued risk of disease exportation to currently unaffected populations. Our model also predicted that populations respond much more dramatically to the introduction of a small vaccine adverse event risk than they do to the introduction of a small infection adverse event risk (Figs 2 and 3), especially for scale-free networks. Moreover, the model predicts that when individuals use global information to estimate perceived cost, the vaccine coverage is higher than when individuals use local information, regardless of whether those perceived costs are accurate or not. Because scale-free networks represent the structure of online social media networks through which debates about vaccine safety are increasingly channeled^72–74^, our results suggest that growing use of social media networks to obtain information about vaccines and infectious diseases could have a net negative effect on vaccine coverage. This suggests an important role for public health dissemination of information about global infection prevalence in populations, such as through Canada’s Fluwatch program^75^, CDC FluView^76^, and mass media.

Although the present study provides a useful framework for understanding how the adverse events from vaccination or infection affect individual vaccination choices, the model was built with simplifying assumptions that could impact model predictions. For instance, we assume that all the dynamics such as transmission of infection, information of adverse events, and individuals’ vaccination dynamics propagate on same network. In reality this may not be true: the infection network of a population can be very different from the social or other networks through which information and opinions spread. Second, we also assume that the network is static, However, real-world networks can evolve over the timescales of interest, and sometimes in response to infection dynamics. The degree of impact of adverse events either from vaccination or infection may also depend nonlinearly on the number of events occurring, but here we assumed a simple additive relationship that may not apply under all conditions in real populations. This presents scope for future research. Vaccine effectiveness is another potential important factor that can influence decisions-making. It has been shown by previous research that imperfect vaccines can generate nontrivial dynamics. For instance, beyond a certain point, increasing the vaccine efficacy can cause a decrease in the proportion of individuals who seek vaccination^77,78^. Our work may be improved by incorporating this important aspect of vaccination decision-making.

The present study suggests that individual vaccinating decisions respond differently to rare but severe vaccine adverse events, than to common but mild events, and that certain network types (such as scale-free networks) are particularly vulnerable to the harmful effects of false stories about severe vaccine adverse events. Models allow us to tease apart the influence of different potential mechanisms, and to explore how coupled behavior-disease systems will respond to different interventions. Thus, public health can use such models to increase vaccine acceptance. For instance, strategies of spreading knowledge through social networks about vaccine preventable diseases, using dramatic narratives and pictures to communicate disease risk, and correcting misconceptions and myths about vaccines^79–81^ could provide a counterweight to the effects of false vaccine risks spreading through scale-free social networks.

## Supplementary information

Supplementary Information

Supplementary Code
