# Supplementary material for: The impact of rare but severe vaccine adverse events on behaviour-disease dynamics: a network model
Source: Sci Rep. 2019 May 9;9:7164. doi: 10.1038/s41598-019-43596-7 (PMC6509123; doi:10.1038/s41598-019-43596-7)
Supplement: Supplementary file 1 — Supplementary Information [file 41598_2019_43596_MOESM1_ESM.pdf]

Supplementary Material
The impact of rare but severe vaccine adverse events on behaviour-disease dynamics: a network model

Samit Bhattacharyya[*1], Amit Vutha[†2], and Chris T. Bauch[‡3]

[1]Mathematics, School of Natural Sciences, Shiv Nadar University, India
[2]ICTS, Tata Institute for Fundamental Research, India
[3]Department of Applied Mathematics, University of Waterloo, Canada

April 16, 2019

[*]Corresponding author: Tel: +91 120 3819100 (Extn. 136), Email: samit.b@snu.edu.in
[†]amitchandrav@gmail.com
[‡]cbauch@uwaterloo.ca

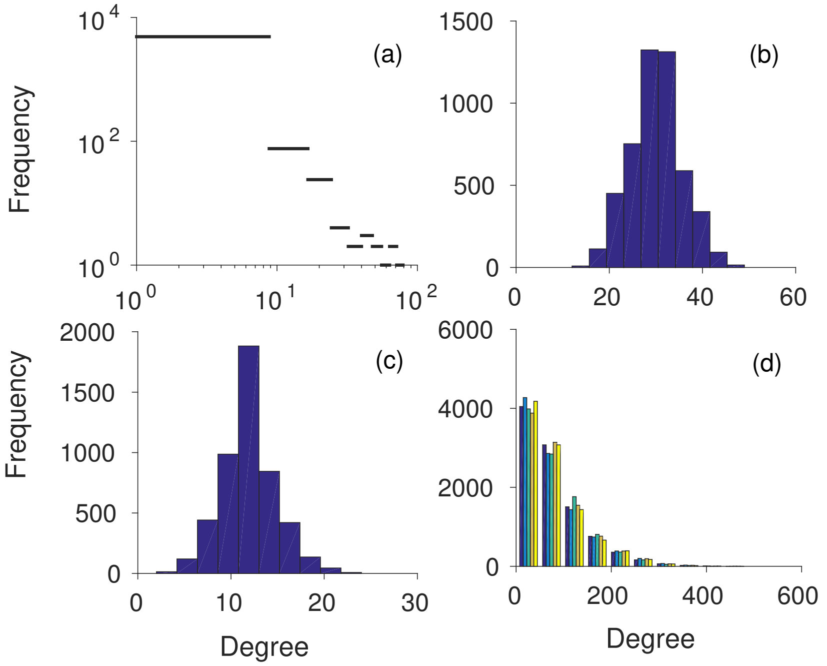

Figure S1: Degree distribution of (a) Power law (scale free) network, (b) Erdos-Renyi random network, (c) Small world network, (d) all five empirical network from Portland, Oregon.

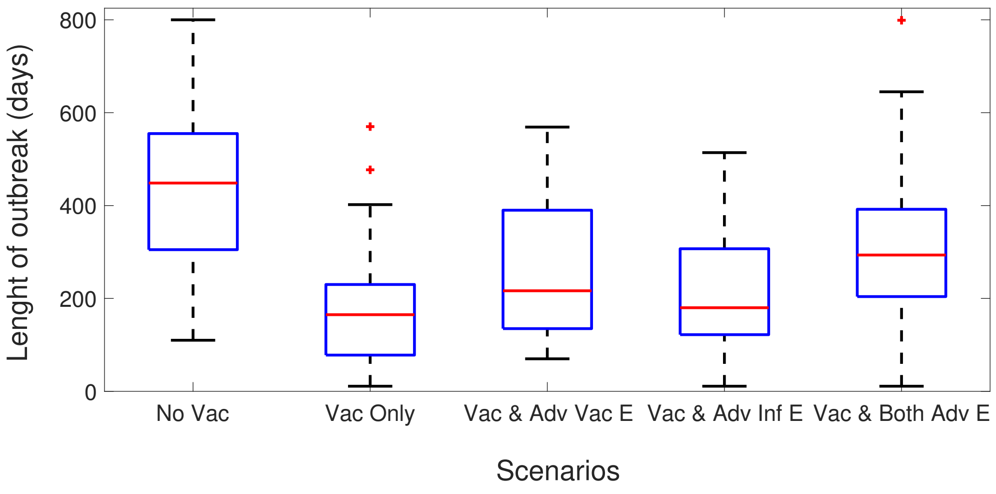

Figure S2: Statistics of length of outbreak tails under five different scenarios (ref: Figure 1 in main text). Same baseline parameter values have been used as in Figure 1 in the main text for all simulations.

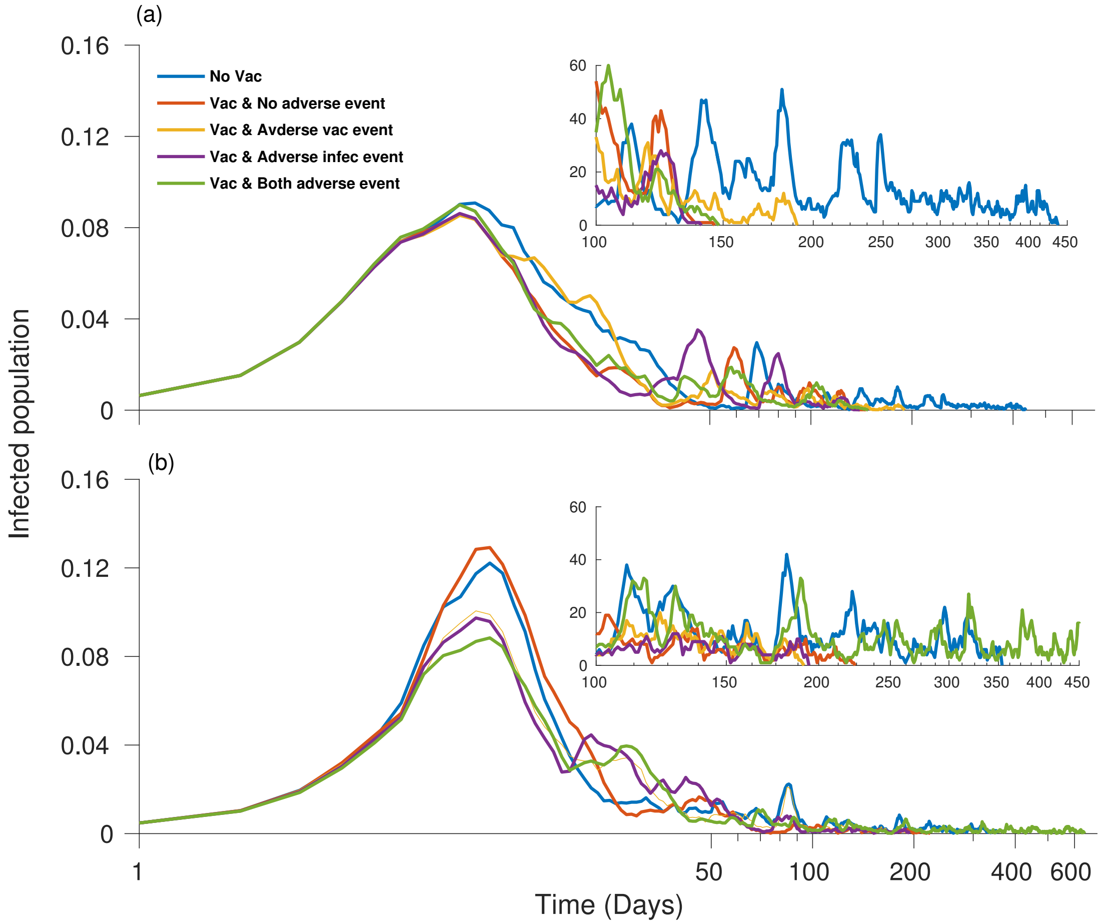

Figure S3: Sample time series plot showing the proportion infected population under different scenario (ref: Figure 1 in main text): (a) when individual perceived risk of infection is based only on global information ($\rho = 0$), and (b) when individual perceived risk of infection is based only on local information ($\rho = 1$).

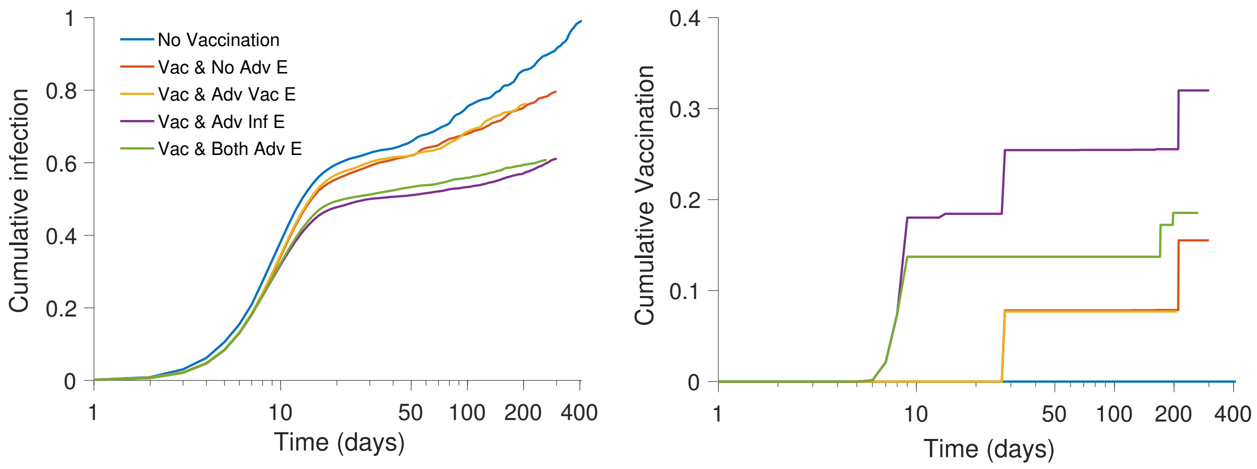

Figure S4: Sample time series plot showing the proportion infected population and vaccinated population under same baseline parameter values as in Figure 1 in main text.

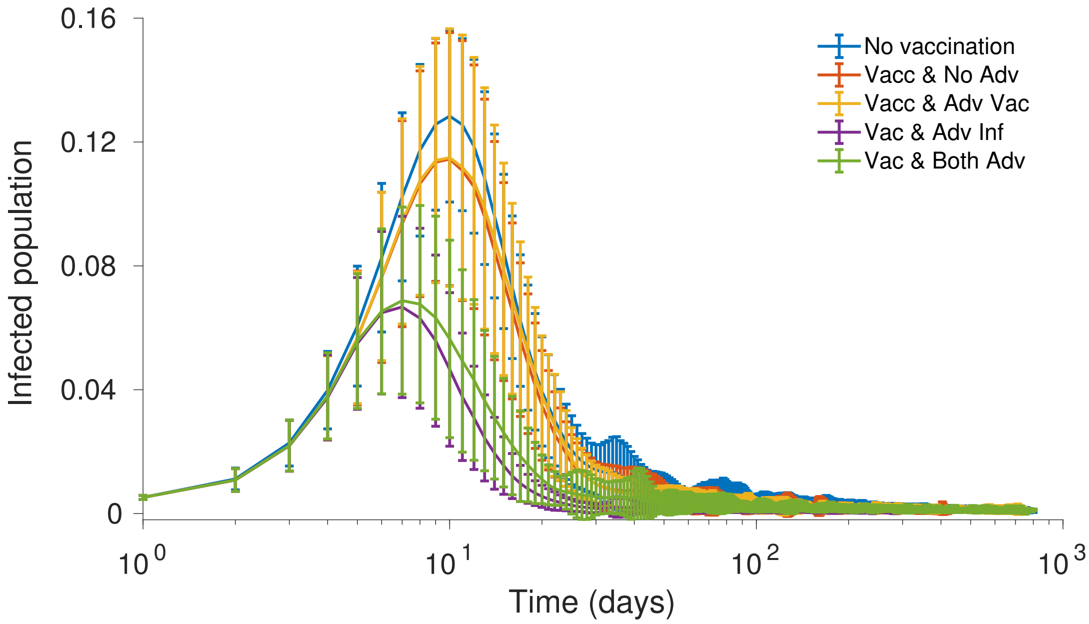

Figure S5: Plot of the proportion of infected population under different scenario, when the information and effect of adverse events disseminated globally ($\omega = \nu = 1$). Parameters values are same as in Figure 1 in main text.

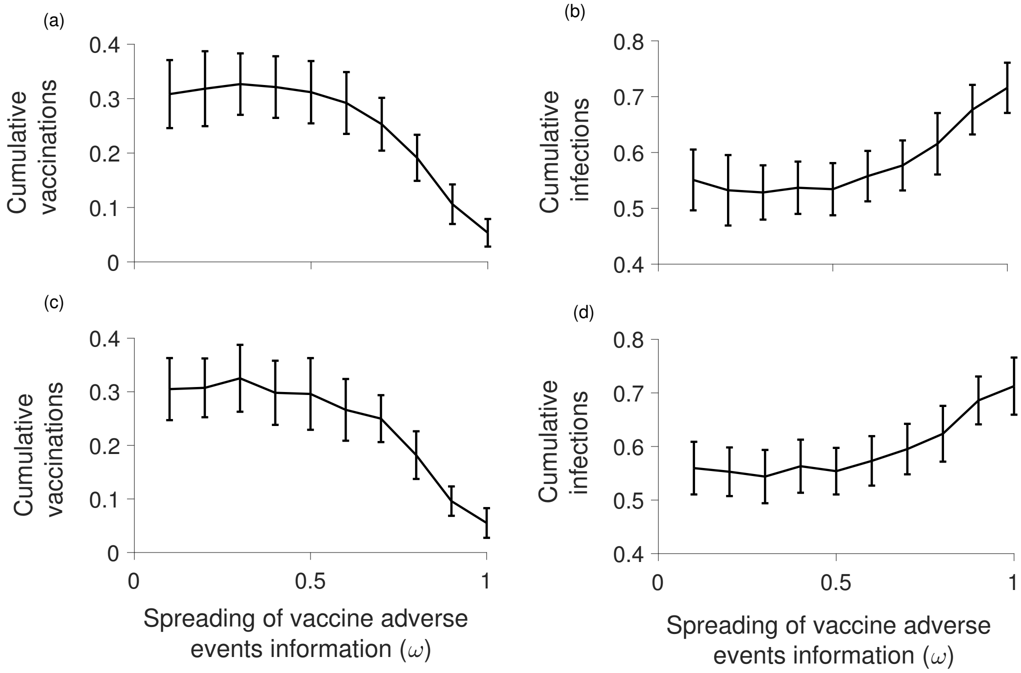

Figure S6: Plot of cumulative vaccinations and cumulative infections under different values of spreading coefficient (ω) of vaccine adverse events when spreading of infection adverse events parameter (a - b) $\nu = 0.25$ and (c - d) $\nu = 0.5$. Other parameters values are same as in Figure 1 in the main text.

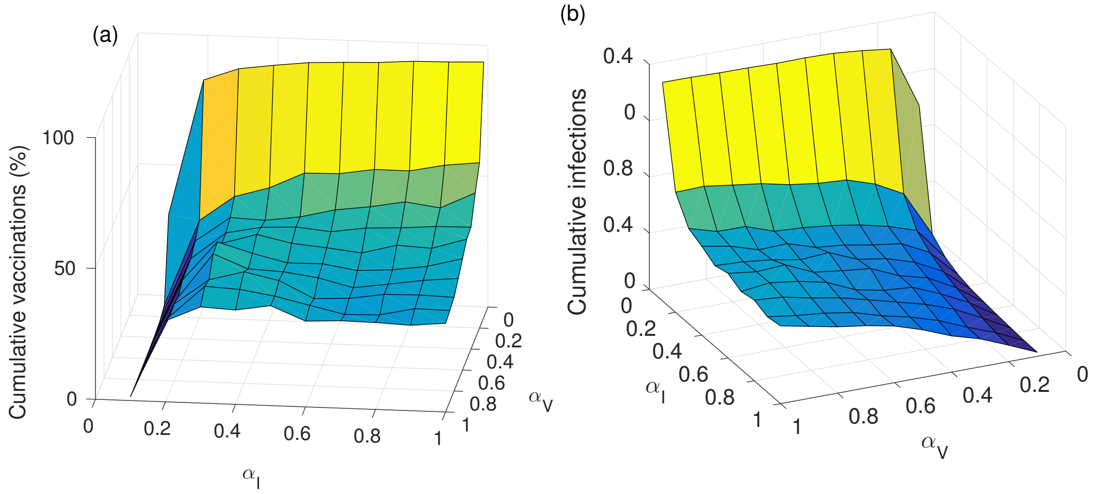

Figure S7: Average (a) cumulative proportion of vaccinated and (b) cumulative proportion of infected at different values of α_V and α_I.

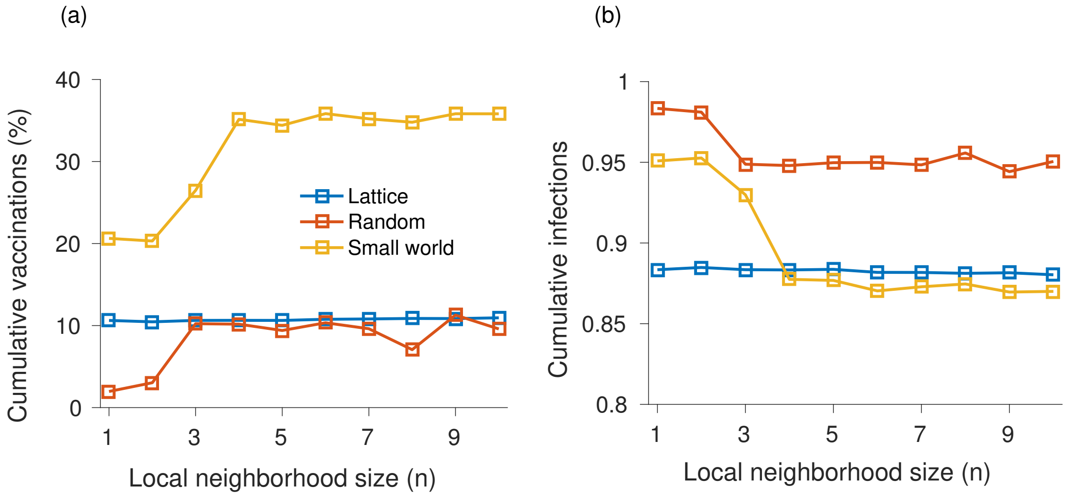

Figure S8: Change in (a) cumulative proportion of vaccinated and (b) cumulative proportion of infected with change in local neighborhood size (n) for different networks.

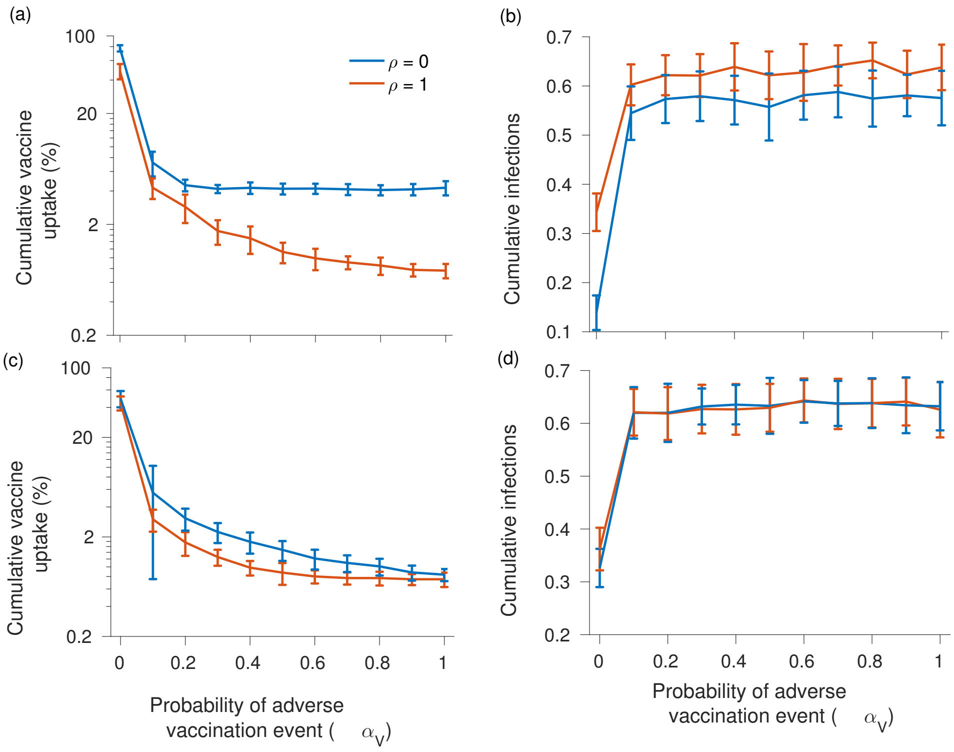

Figure S9: Figure shows (a) cumulative proportion of vaccinated and (b) cumulative proportion of infected with definitions of the perceived risk of infection as in equation (5). Similarly, (c) cumulative vaccination coverage and (b) cumulative infections with definitions of the perceived risk of infection as in equation (6). $\rho = 1$ means individual perceived risk of infection completely depends on local information, whereas $\rho = 0$ is that on completely global information.

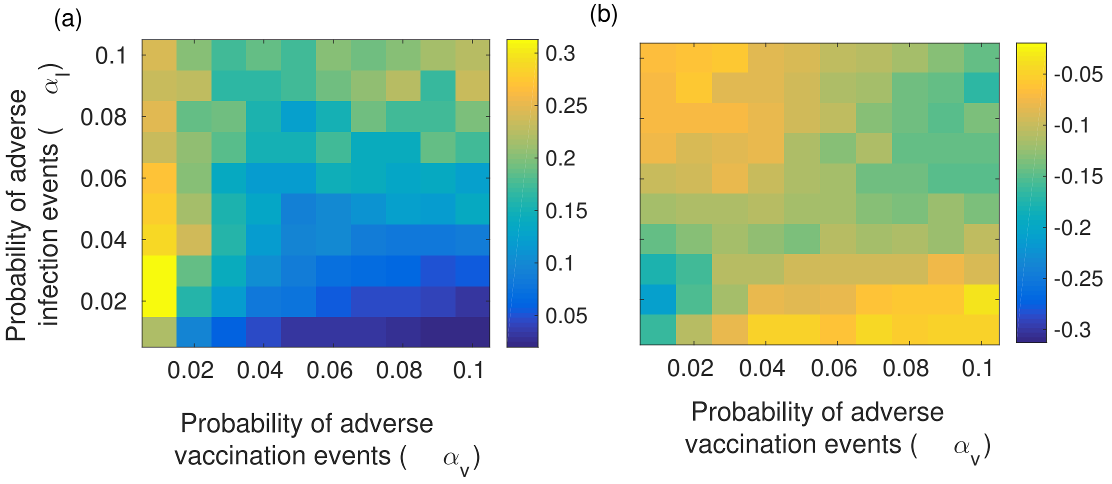

Figure S10: Figure shows the difference in (a) cumulative proportion of vaccinated and (b) cumulative proportion of infected between $\rho = 0$ and $\rho = 1$ in the definition of the perceived risk of infection by equations (5) and (6) (refer Figure S9). Note that the scale of figure (b) is in negative values, means infection in case of $\rho = 1$ is higher than that of $\rho = 0$.

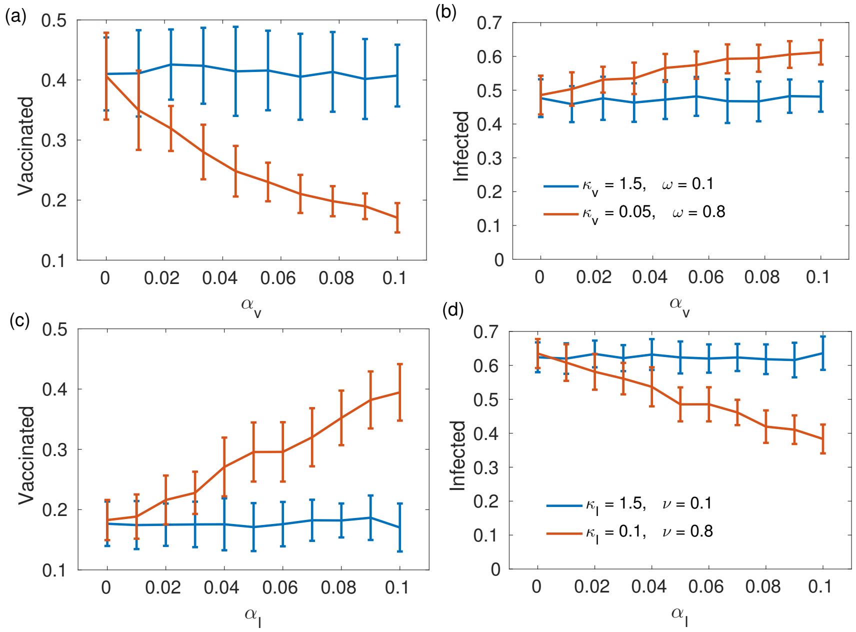

Figure S11: Figure shows (a) cumulative proportion of vaccinated and (b) cumulative proportion of infected for higher and lower values of κ_V and ω at different probability of adverse vaccination events α_V. Similarly (c) and (d) depicts same for higher and lower values of κ and ν at different probability of adverse infection events α_I. This figure shows that less severe events have more impact on the vaccination coverage dynamics in network compare to highly severe adverse events if the information spread rapidly.
