# Supplementary material for: The impact of rare but severe vaccine adverse events on behaviour-disease dynamics: a network model
Source: Sci Rep. 2019 May 9;9:7164. doi: 10.1038/s41598-019-43596-7 (PMC6509123; doi:10.1038/s41598-019-43596-7)
Supplement: Supplementary file 2 — Supplementary Code [file 41598_2019_43596_MOESM2_ESM.pdf]

Supplementary Material
(Simulation code)
The impact of rare but severe vaccine adverse events on behaviour-disease dynamics: a network model

Samit Bhattacharyya*[1], Amit Vutha†[2], and Chris T. Bauch‡[3]

[1]Mathematics, School of Natural Sciences, Shiv Nadar University, India
[2]ICTS, Tata Institute for Fundamental Research, India
[3]Department of Applied Mathematics, University of Waterloo, Canada

March 20, 2019

*Corresponding author: Tel: +91 120 3819100 (Extn. 136), Email: samit.b@snu.edu.in
†amitchandrav@gmail.com
‡cbauch@uwaterloo.ca

```
/*
 * The following is C code for simulations to accompany “The impact of rare but
severe vaccine adverse
 * events on behaviour-disease dynamics: a network model” by Bhattacharyya et.
al.
 * ----------------------
 * The following program uses the igraph library for c, which must
 * be installed prior to compilation
 *
 * Details can be found at https://igraph.org/c/
 * ----------------------
 * Dependencies:
 *
 * - Network file stored in PAJEK format (filename specified as graphFilename)
 * - Parameters for dynamics (filename specified as parametersFilename), can be
saved as .csv file.
 *
 * Output files:
 * - Counts of vaccinations and infections for each timestep, stored into
 * .csv files specified under OutputFilename
 *
 */

/* -------------------------Include some libraries */

#include <igraph.h>
#include <stdio.h>
#include <math.h>
#include <time.h>
#include <vector>
#include <fstream>
#include <iostream>
#include <string.h>

using namespace std;

/* --------------  Names of parameters as strings to display to user */

char parameterNames[19][30] = {"alphaVaccinate", "alphaInfect",
"kappaVaccinate",
            "kappaInfect", "omegaVaccinate", "omegaInfect",
            "numberOfInfectedNodes", "SIRBeta", "SIRGamma",
            "rho", "L", "fermiBeta",
            "birthDeathRate", "caseImportRate", "vacMemory",
            "disMemory", "doubleCaseImport", "vaccinateYes",
            "localDist"};

/* --------- Initialize global variables for parameters and counters */
```

```
float alphaVaccinate, alphaInfect, kappaVaccinate, kappaInfect, omegaVaccinate,
omegaInfect,
SIRBeta, SIRGamma, rho, L, fermiBeta, birthDeathRate, caseImportRate;
int numberOfInfectedNodes, vacMemory, disMemory, doubleCaseImport,
vaccinateYes, localDist;
int sirCounter;
int sizeOfGraph;
int infectedNodesVector[20];
char graphFilename[40]="ScaleFreeGraph5000_A.net";
char parametersFilename[40]="parameters.csv";
char outputFilename[2][40]={"Infections.csv","Vaccinations.csv"};
char outputFilenameTimestamped[40];
char startTime[40];
char endTime[40];

/* ------------------------------------ Some global igraph variables */

/* Store the graph */
igraph_t graph;

/* Store the distance matrix */
igraph_matrix_t shortestPath;


/* 5 copies of some variables for 5 different scenarios */

/* Store occurences of vaccinations and infections at nodes to enforce memory */
igraph_vector_t vacTracker[5];
igraph_vector_t disTracker[5];

/* Initial disease state - randomly allotted for each run */
igraph_vector_t initDiseaseState[5];

/* Store the SIRV state of each node for current and previous step */
igraph_vector_t diseaseState[5];
igraph_vector_t priorDiseaseState[5];

/* Store perceived vaccination and infection risks for each node */
igraph_vector_t perceivedVaccineRisk[5];
igraph_vector_t perceivedInfectionRisk[5];

/* Counters for various events */
int cumulativeNewInfections[5];
int cumulativeCaseImportations[5];
int cumulativeVaccinations[5];
int advVacCounter[5];
int advInfCounter[5];
int vacCounter[5];
```

```
/* Enforces flags to distinguish between different scenarios, 1 - yes */
int newVaccinateYes[5]={0,1,1,1,1};
int adverseVacYes[5]={0,0,1,0,1};
int adverseInfYes[5]={0,0,0,1,1};

/* Counter to decide stopping points for simulations */
float reachedZeroDiseases[5]={0,0,0,0,0};
int SIRStepsTotal[5]={0,0,0,0,0};

/* Reads parameter values for a .csv file*/
void readParametersFromFile()
{
	std::ifstream parametersFile;
	parametersFile.open(parametersFilename);

	parametersFile
	>> alphaVaccinate >> alphaInfect >> kappaVaccinate >> kappaInfect
	>> omegaVaccinate >> omegaInfect >> numberOfInfectedNodes
	>> SIRBeta >> SIRGamma
	>> rho >> L >> fermiBeta
	>> birthDeathRate >> caseImportRate
	>> vacMemory >> disMemory
	>> doubleCaseImport >> vaccinateYes >> localDist ;
	parametersFile.close();
}

/* Dispays the result of reading parameters to screen for users*/
void displayParametersToScreen()
{
	printf("---------------------------------------------\n");
	printf("------------------------- SIMULATION PARAMETERS\n");
	printf("------------------------------------------------\n");
	printf("1) alphaVaccinate:   %f \n", alphaVaccinate);
	printf("2) alphaInfect:         %f \n", alphaInfect);
	printf("3) kappaVaccinate:  %f \n", kappaVaccinate);
	printf("4) kappaInfect:        %f \n", kappaInfect);
	printf("5) omegaVaccinate:  %f \n", omegaVaccinate);
	printf("6) omegaInfect:       %f \n", omegaInfect);
	printf("----------------------------------------------\n");
	printf("7) numberOfInfectedNodes: %i \n", numberOfInfectedNodes);
	printf("8) SIRBeta:                 %f \n", SIRBeta);
	printf("9) SIRGamma:                        %f \n", SIRGamma);
	printf("10) rho:                    %f \n", rho);
	printf("11) L:                      %f \n", L);
	printf("12) fermiBeta:                      %f \n", fermiBeta);
	printf("----------------------------------------------\n");
	printf("13) birthDeathRate:             %f \n", birthDeathRate);
	printf("14) caseImportRate:             %f \n", caseImportRate);
```

```
	printf("15) vacMemory:			%i \n", vacMemory);
	printf("16) disMemory:			%i \n", disMemory);
	printf("17) doubleCaseImport:	%i \n", doubleCaseImport);
	printf("18) vaccinateYes:	%i \n", vaccinateYes);
	printf("------------------------------------------------\n");
	printf("19) localDist:		%i \n", localDist);
	printf("---------------------------------------------------------------------------------
---------------------------------------------\n");
}

/* Routines to record starting and end times to display to user and timestamping
*/
void recordStartTime()
{
	struct tm *timed;
	time_t now = time(NULL);
	timed = localtime(&now);
	strftime(startTime, sizeof(startTime), "(%d/%m/%Y) %H:%M:%S",
timed);
}

void recordEndTime()
{
	struct tm *timed;
	time_t now = time(NULL);
	timed = localtime(&now);
	strftime(endTime, sizeof(endTime), "(%d/%m/%Y) %H:%M:%S",
timed);
}

/* Function: Calculate a distance matrix of shortest paths between pairs of nodes
*/
void calculate_shortestpath_matrix(igraph_t *g)
{
   igraph_matrix_init(&shortestPath, 1, 1);
   igraph_vs_t allVertices;
   igraph_vs_all(&allVertices);
	igraph_shortest_paths(g, &shortestPath,allVertices,allVertices,
IGRAPH_ALL);
   igraph_vs_destroy(&allVertices);
}

/* Function: Infect some vertices randomly, in this case "n"-- specified when
 * function is called */

void assign_disease_states(int n, int size)
{
	int randomVertex=0;
	printf("\n Assigned vertices:  ");
```

```
	for (int i=0; i<n; i++)
	{
		randomVertex=rand() % size;
		for (int j=0;j<5; j++)
		{
			VECTOR(initDiseaseState[j])[randomVertex]=1;
		}
		printf("%i, ", randomVertex);
		infectedNodesVector[i]=randomVertex;
	}
	printf("\n -------------------------------------\n");
}

/* ---------------------------- Inidividual routines to count S-I-R-V */

/* Function: Total number of susceptible states*/
int count_susceptible_states(igraph_vector_t *v)
{
	int count=0;
	for (int i=0; i<igraph_vector_size(v); i++)
	{
		if(VECTOR(*v)[i]==0) count++;
	}
	return count;
}

/* Function: Total number of diseased states*/
int count_disease_states(igraph_vector_t *v)
{
	int count=0;
	for (int i=0; i<igraph_vector_size(v); i++)
	{
		if(VECTOR(*v)[i]==1) count++;
	}
	return count;
}

/* Function: Total number of recovered states*/
int count_recovered_states(igraph_vector_t *v)
{
	int count=0;
	for (int i=0; i<igraph_vector_size(v); i++)
	{
		if(VECTOR(*v)[i]==2) {count++;}
	}
  return count;
}

/* Function: Total number of vaccinated states*/
```

```
int count_vaccinated_states(igraph_vector_t *v)
{
	int count=0;
	for (int i=0; i<igraph_vector_size(v); i++)
	{
		if(VECTOR(*v)[i]==3) {count++;}
	}
	return count;
}

/* Function: Compute number of diseased Neighbours */
int count_infected_Neighbours(igraph_vector_t *v, int vertex)
{
	int numberOfInfectedNeighbours=0;
	igraph_vector_t vertexNeighbours;
	igraph_vector_init(&vertexNeighbours,10);
	igraph_neighbors(&graph, &vertexNeighbours,vertex, IGRAPH_ALL);
   for (int i=0; i<igraph_vector_size(&vertexNeighbours); i++)
   {
		if(VECTOR(*v)[(int) VECTOR(vertexNeighbours)[i]]==1)
numberOfInfectedNeighbours++;
	}
	igraph_vector_destroy(&vertexNeighbours);
	return numberOfInfectedNeighbours;
}

/* Function: Shortest distance calculator */
int shortest_pathlength(int vec1, int vec2)
{
	int dist=MATRIX(shortestPath, vec1, vec2);
   return dist;
}

/* Function: Compute number of local diseased Neighbours */
int count_local_infected_Neighbours(igraph_vector_t *v, int vertex)
{
	int count=0;
	for (int i=0; i<igraph_vector_size(v); i++)
	{
		double dist=(double)shortest_pathlength(i,vertex);
		if((VECTOR(*v)[i]==1) && (dist<=localDist)) count++;
	}
	return count;
}

/* Function: Compute number of local Neighbours */
int count_local_Neighbours(int vertex)
{
	int count=0;
```

```
	for (int i=0; i<sizeOfGraph; i++)
	{
		double dist=(double)shortest_pathlength(i,vertex);
		if(dist<=localDist) count++;
	}
	return count;
}

/* Function: Random between 0 and 1 drawn from a uniform distribution */
float random_zero_one()
{
	return static_cast <float> (rand()) / static_cast <float> (RAND_MAX);
}

/* Function: Random event with a given probability */
bool random_event(float prob)
{
	float r=random_zero_one();
	if (r<prob) return 1;
	if (r>=prob) return 0;
}

/* Function: SIR Dynamics */
void SIR_Dynamics(float beta, float g)
{
	/* Variables to store random numbers*/
	float r,r1;

	/* Iterate over all nodes */
	for (int i=0; i<sizeOfGraph; i++)
	{
		r = random_zero_one();
		r1 = random_event(alphaInfect);

		/*Repeat for each of the five scenarios */
		for(int jc=0;jc<5;jc++)
		{
			/* Stopping condition */
			if(reachedZeroDiseases[jc]!=1)
			{
				/* Updates disease memory for nodes that haven't
been infected */
				if(VECTOR(disTracker[jc])[i]!=0)
				{
					VECTOR(disTracker[jc])[i]++;
				}
				if(VECTOR(priorDiseaseState[jc])[i]==1)
				{
```

```
					/* Triggers an adverse event associated with
infection */
					if
(r1&&(VECTOR(disTracker[jc])[i]<=disMemory)&&(adverseInfYes[jc]))
					{
						for (int j=0; j<sizeOfGraph; j++)
						{
							double
dist=(double)shortest_pathlength(i,j);
							{

	VECTOR(perceivedInfectionRisk[jc])[j]=VECTOR(perceivedInfectionRisk[
jc])[j]*(1+kappaInfect*pow((double)omegaInfect, (double) dist));
							}
						}
						advInfCounter[jc]++;
					}
					/* Condition for recovery */
					if (r<g)
					{
						VECTOR(diseaseState[jc])[i]=2;
					}
				}
			}
			/* Computes node-wise probability of acquiring an
infection for
			 * susceptible nodes using the function theta*/
			float probOfInfection=(float)(1-pow((double)(1-
beta),(double) count_infected_Neighbours (&priorDiseaseState[jc],i)));
			if
((r<probOfInfection)&&(VECTOR(priorDiseaseState[jc])[i]==0))
			{
				VECTOR(diseaseState[jc])[i]=1;
				VECTOR(disTracker[jc])[i]++;
				cumulativeNewInfections[jc]++;
			}
		}
	}
}

/* Function: Calculate the function theta for a given vertex and a disease state */
float theta(igraph_vector_t *disStat, int vertex)
{
	float thetaValue;
	igraph_vector_t vertexNeighbours;
igraph_vector_init(&vertexNeighbours,10);
	igraph_neighbors(&graph, &vertexNeighbours,vertex, IGRAPH_ALL);
	float localInfections=count_local_infected_Neighbours(disStat, vertex);
	float globalInfections=count_disease_states(disStat)-localInfections;
```

```
	thetaValue=(
	(float)(rho*localInfections/(count_local_Neighbours(vertex))+(1-
rho)*(float) count_disease_states(disStat)/sizeOfGraph));
	return thetaValue;
}

/* Function: Vaccination decision dynamics */
void vaccination_decision()
{
	/* Variables for random number and fermi function */

	float r, fermiValue;
	r=random_zero_one();

	/* Cycle through different scenarios */
	for (int jc=0;jc<5;jc++)
	{
		if(count_susceptible_states(&diseaseState[jc])!=0)
		{
			/* Span through all nodes */
			for (int i=0; i<sizeOfGraph; i++)
			{
				/* Update memories of disease acquisition */
				if(VECTOR(vacTracker[jc])[i]!=0)
				{
					VECTOR(vacTracker[jc])[i]++;
				}

				/* Condition for an adverse infection event */
				if
(random_event(alphaVaccinate)&&(VECTOR(priorDiseaseState[jc])[i]==3)

	&&(VECTOR(vacTracker[jc])[i]<=vacMemory)&&(adverseVacYes[jc]!=0))
				{
					for (int j=0; j<sizeOfGraph; j++)
					{
						double
dist=(double)shortest_pathlength(i,j);
						{

	VECTOR(perceivedVaccineRisk[jc])[j]*=(1+kappaVaccinate*pow((double
)omegaVaccinate,
						(double) dist));
						}
					}
					advVacCounter[jc]++;
				}
				/* Compute the fermi function */
```

```
				fermiValue=1.0/(1+exp(fermiBeta*((float)
VECTOR(perceivedVaccineRisk[jc])[i]-
				(float)
VECTOR(perceivedInfectionRisk[jc])[i]*theta(&diseaseState[jc],i))));

				/* Conditions for vaccination */

	if((r<fermiValue)&&(VECTOR(priorDiseaseState[jc])[i]==0)&&(newVacci
nateYes[jc]!=0))				/* condn for vaccinating */
				{
					VECTOR(diseaseState[jc])[i]=3;
					VECTOR(vacTracker[jc])[i]++;
					cumulativeVaccinations[jc]++;
				}
			}
		}
	}
}

/* Function: Assign initial costs randomly to nodes */
void assign_initial_costs()
{
	float firstRandom, secondRandom;
	for (int i=0; i<sizeOfGraph; i++)
	{
		/* Initial distributions of costs, specified as uniform intervals*/
		firstRandom=0.7+0.3*random_zero_one();
		secondRandom=0.5*random_zero_one();
		for (int j=0;j<5;j++)
		{
			VECTOR(perceivedVaccineRisk[j])[i]= firstRandom;
			VECTOR(perceivedInfectionRisk[j])[i]= secondRandom;
		}
	}
}

/* Function: Birth Death Process */
void birthDeathProcess()
{
	/* Choose a random node in the network */
	int randomVertex=0;
	randomVertex=rand() % sizeOfGraph;

	/* Cycle through different scenarios */
	for(int j=0;j<5;j++)
	{
		if(reachedZeroDiseases[j]!=1)
		{
			VECTOR(diseaseState[j])[randomVertex]=0;
```

```
                    VECTOR(vacTracker[j])[randomVertex]=0;
                    VECTOR(disTracker[j])[randomVertex]=0;
                }
        }
}

/* Function: Case importation process */
void caseImportProcess()
{
        /* Cycle through different scenarios */
        for(int j=0;j<5;j++)
        {
                if(reachedZeroDiseases[j]!=1)
                {
                        int randomVertex=0;
                        /* Code fragment to choose randomly from susceptible
nodes only */
                        int susceptibleVertices[sizeOfGraph];
                        int susceptibleVertexCounter=0;
                        for(int l=0; l<sizeOfGraph; l++)
                        {
                                if (VECTOR(diseaseState[j])[l]==0)
                                {

        susceptibleVertices[susceptibleVertexCounter]=l;
                                        susceptibleVertexCounter++;
                                }

                        }
                        if(count_susceptible_states(&diseaseState[j])>0)
                        {
                                int randomVertexPosition=rand() %
susceptibleVertexCounter;

        randomVertex=susceptibleVertices[randomVertexPosition];
                                VECTOR(diseaseState[j])[randomVertex]=1;
                                VECTOR(vacTracker[j])[randomVertex]=0;
                                VECTOR(disTracker[j])[randomVertex]=1;
                                cumulativeCaseImportations[j]++;
                        }
                        if (random_event(caseImportRate)&& doubleCaseImport)
                        {
                                int susceptibleVertexCounter=0;
                                for(int l=0; l<sizeOfGraph; l++)
                                {
                                        if (VECTOR(diseaseState[j])[l]==0)
                                        {

        susceptibleVertices[susceptibleVertexCounter]=l;
```

```
                                        susceptibleVertexCounter++;
                                }
                        }
                        if(count_susceptible_states(&diseaseState[j])>0)
                        {
                                int randomVertexPosition=rand() %
susceptibleVertexCounter;

        randomVertex=susceptibleVertices[randomVertexPosition];
                                VECTOR(diseaseState[j])[randomVertex]=1;
                                VECTOR(vacTracker[j])[randomVertex]=0;
                                VECTOR(disTracker[j])[randomVertex]=1;
                                cumulativeCaseImportations[j]++;
                        }
                }
        }
    }
}

void readGraph()
{
        /* Set up an attribute table - used to assign colours */
        igraph_i_set_attribute_table(&igraph_cattribute_table);

        /* Read graph from Pajek file */
        FILE *ifile;
        ifile=fopen(graphFilename, "r");
        igraph_read_graph_pajek(&graph, ifile);
        fclose(ifile);

        /* Computes a distance matrix */
        calculate_shortestpath_matrix(&graph);
}

/* Function: Calls SIR and vaccination dynamics, records information to files */
void networkDynamics()
{
        /* Globally records the size of the inputted graph */
        sizeOfGraph=igraph_vcount(&graph);

        /* Sets initial counters =0 for each run of the network dynamics */
        for(int i=0;i<5;i++)
        {
                cumulativeNewInfections[i]=0;
                cumulativeCaseImportations[i]=0;
                cumulativeVaccinations[i]=0;
                advVacCounter[i]=0;
                advInfCounter[i]=0;
                vacCounter[i]=0;
```

```
			igraph_vector_init(&diseaseState[i],(long int)
igraph_vcount(&graph));
			igraph_vector_init(&priorDiseaseState[i],(long int)
igraph_vcount(&graph));
			igraph_vector_init(&vacTracker[i],(long int)
igraph_vcount(&graph));
			igraph_vector_init(&disTracker[i],(long int)
igraph_vcount(&graph));
			igraph_vector_fill(&diseaseState[i],0);
			igraph_vector_fill(&priorDiseaseState[i],0);
			igraph_vector_fill(&vacTracker[i],0);
			igraph_vector_fill(&disTracker[i],0);
			igraph_vector_copy(&diseaseState[i], &initDiseaseState[i]);
			igraph_vector_init(&perceivedVaccineRisk[i],(long int)
igraph_vcount(&graph));
			igraph_vector_init(&perceivedInfectionRisk[i],(long int)
igraph_vcount(&graph));
		}

		/* This functions allots values randomly b/w 0 and 1 */
		assign_initial_costs();

		/* Counter for step number */
		sirCounter=0;
		for(int j=0; j<5;j++)
		{
			igraph_vector_copy(&priorDiseaseState[j], &diseaseState[j]);
		}
		int combinedReached=0;

		printf("\n Running SIR Dynamics...\n ");

		/* Open a CSV file to enter disease states*/
		std::ofstream outputFile1;
		outputFile1.open(outputFilename[0]);
		std::ofstream outputFile2;
		outputFile2.open(outputFilename[1]);

		outputFile1<< "No Vac."
			<<","<< "Vac+ No Adv"
			<<","<<"Vac +Adv Vac only"
			<<","<<"Vac + Adv Inf only"
			<<","<<"Vac + Adv Vac+Adv Inf"
			<<"\n";

		outputFile2<< "No Vac."
		<<","<< "Vac+ No Adv"
		<<","<<"Vac +Adv Vac only"
		<<","<<"Vac + Adv Inf only"
```

```
	<<","<<"Vac + Adv Vac+Adv Inf"
	<<"\n";

	/* Stopping conditions*/
	while((sirCounter<1000)&&(combinedReached!=1))
	{
		/* Update step counters */
		sirCounter++;

		/* Run SIR dynamics */
		SIR_Dynamics(/* beta =*/SIRBeta, /* gamma = */  SIRGamma);

		/* Update vaccination decisions and possible adverse vaccination
events */
		vaccination_decision();

		/* Cycle through and display scenarios  */
		for (int j=0; j<5;j++)
		{
			SIRStepsTotal[j]++;
			switch(j)
			{
				case 0: printf("\n (No vaccinations) ");break;
				case 1: printf("\n (Vaccine+ No adv inf + No adv vac)
");break;
				case 2: printf("\n (Vaccine + No adv inf + adv vac)
");break;
				case 3: printf("\n (Vaccine + adv inf + no adv vac)
");break;
				case 4: printf("\n (Vaccine + adv inf +  adv vac)
");break;
			}

			/* Trasnfer current disease states to a separate location for
next step */
			igraph_vector_copy(&priorDiseaseState[j],
&diseaseState[j]);

			/* Output into csv files if dynamics are still running */
			if(reachedZeroDiseases[j]!=1)
			{

	reachedZeroDiseases[j]=(count_disease_states(&diseaseState[j])==0);
				SIRStepsTotal[j]++;

	outputFile1<<count_disease_states(&diseaseState[j])<<",";

	outputFile2<<count_vaccinated_states(&diseaseState[j])<<",";
			}
```

```
                else
                {
                        outputFile1<<",";
                        outputFile2<<",";
                }
            }
            outputFile1<<"\n";
            outputFile2<<"\n";

            /* Evaluate a combined stoppping condition for all scenarios */
            combinedReached=(reachedZeroDiseases[0] &&
reachedZeroDiseases[1] && reachedZeroDiseases[2] &&
reachedZeroDiseases[4] && reachedZeroDiseases[4]);

            /* Birth death proces */
            if (random_event(birthDeathRate))

            {
                        birthDeathProcess();
            }

            /* Case importation process */
            if (random_event(caseImportRate))
            {
                caseImportProcess();
            }
        }

        /* Close output streams */
  outputFile1.close();
  outputFile2.close();
  printf("Output files created: %s and %s", outputFilename[0],
outputFilename[1]);
}

int main(void)
{
        recordStartTime();
        readParametersFromFile();
        displayParametersToScreen();
        readGraph();


/* Introduce infections in some nodes by changing diseaseState,
 * numberOfInfectedNodes can be set globally */
        srand (time(NULL));
        for(int i=0; i<5;i++)
        {
```

```
			igraph_vector_init(&initDiseaseState[i],(long int)
igraph_vcount(&graph));
			igraph_vector_fill(&initDiseaseState[i],0);
		}
		printf("\n Assigning disease to %i nodes...\n ", numberOfInfectedNodes);
		assign_disease_states(numberOfInfectedNodes,igraph_vcount(&graph));

		/* One iteration of network dynamics */
		networkDynamics();

		/* Destroy variables and free up memory */
		for (int i=0;i<5;i++)
		{
			igraph_vector_destroy(&diseaseState[i]);
			igraph_vector_destroy(&initDiseaseState[i]);
			igraph_vector_destroy(&vacTracker[i]);
			igraph_vector_destroy(&disTracker[i]);
			igraph_vector_destroy(&perceivedInfectionRisk[i]);
			igraph_vector_destroy(&perceivedVaccineRisk[i]);
			igraph_vector_destroy(&priorDiseaseState[i]);
		}

		igraph_destroy(&graph);

		/* Wrap things up */
		recordEndTime();
		printf("\n Start time: %s \n ", startTime);
		printf("\n End time: %s \n ", endTime);
		return 0;
}
```
